# Characterization of neuronal intrinsic properties and synaptic transmission in layer I of anterior cingulate cortex from adult mice

**DOI:** 10.1186/1744-8069-8-53

**Published:** 2012-07-20

**Authors:** Xiang-Yao Li, Tao Chen, Giannina Descalzi, Kohei Koga, Shuang Qiu, Min Zhuo

**Affiliations:** 1 Center for Neuron and Disease, Frontier Institute of Science and Technology, Xi’an Jiaotong University, Xi’an 710054, China; 2 Department of Physiology, Faculty of Medicine, University of Toronto, Medical Science Building, Room #3342, 1 King’s College Circle, Toronto, Ontario, M5S 1A8, Canada

**Keywords:** Spontaneous firing neurons, Layer I, Adult mice, Anterior cingulate cortex, Kainate receptor

## Abstract

The neurons in neocortex layer I (LI) provide inhibition to the cortical networks. Despite increasing use of mice for the study of brain functions, few studies were reported about mouse LI neurons. In the present study, we characterized intrinsic properties of LI neurons of the anterior cingulate cortex (ACC), a key cortical area for sensory and cognitive functions, by using whole-cell patch clamp recording approach. Seventy one neurons in LI and 12 pyramidal neurons in LII/III were recorded. Although all of the LI neurons expressed continuous adapting firing characteristics, the unsupervised clustering results revealed five groups in the ACC, including: Spontaneous firing neurons; Delay-sAHP neurons, Delay-fAHP neurons, and two groups of neurons with ADP, named ADP1 and ADP2, respectively. Using pharmacological approaches, we found that LI neurons received both excitatory (mediated by AMPA, kainate and NMDA receptors), and inhibitory inputs (which were mediated by GABA_A_ receptors). Our studies provide the first report characterizing the electrophysiological properties of neurons in LI of the ACC from adult mice.

## Introduction

Knowledge about the intrinsic properties of neurons helps to reveal the neuronal mechanisms of brain functions [1,2]. The mammalian neocortex is responsible for different brain functions, such as sensory perception and cognitive function [3-7]. The cortical network is mainly consisted with projection glutamatergic neurons and local GABAergic interneurons, the projection neurons communicate with other brain areas by extending axons to distant brain targets, interneurons modulate the temporal demands of brain functions by supporting inhibitory components [2]. Cumulative studies have found that the firing patterns, molecular expression profiles and innervations targets of the interneurons are with rich diversity, which is involved in the modulation of neuronal network [8]. Therefore, knowing the diversity of interneurons of different brain areas will be necessary for the understanding of brain functions.

**Figure 1:**
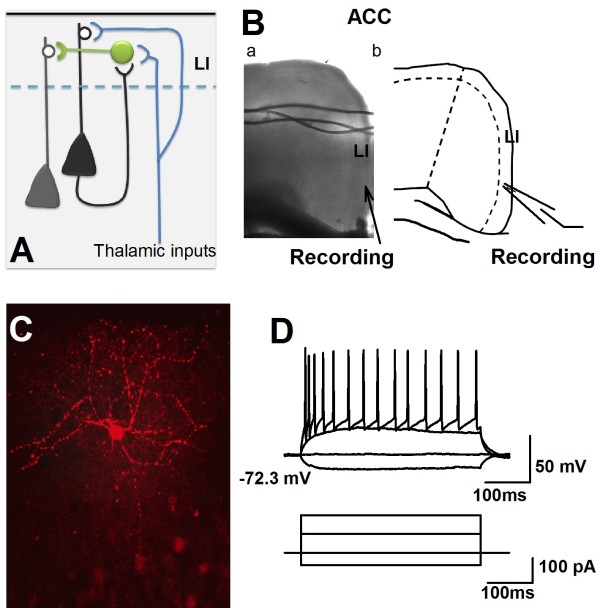
**Whole-cell patch-clamp recording were performed in the LI of the ACC. A**), Diagram representing the involvement of LI neurons to the cortical network. **B**), a, Representative coronal section showed the performing of whole-cell patch clamp recording on an ACC slice. Bb, Schematic diagram represented the location of recording in LI of the ACC. **C**), Photomicrograph of an example LI neurons labeled with biocytin. **D**), Representative showed the APs firing of a recorded LI neuron under current injection.

Layer I (LI) is a unique part of the neocortex which has less cell density but a high percentage of GABAergic neurons, and receives strong dendritic tuft branches from other layers. The functions of cortical LI are age dependent. For example, during early stages of neocorticogenesis, the Cajal-Retzius cells in LI provide the signaling for neuronal migration [9]. After cortical development, LI neurons are involved in the modulation of cortical network by providing inhibition (Figure1A) [2]. LI neurons receive projections from both local area [10,11] and a large population of thalamocortical neurons [12-15]. It has been proposed that cortical LI neurons play a special role in top-down synaptic interaction within cortical networks [14-19]. Following the development of modern genetic techniques, mice have been broadly used to study the functions of interneurons in the central nervous system [2,20,21]. However, characterizations of LI neurons from adult mice have not been reported (see Table1). 

**Table 1:** Summary of previous works on the electrophysiological and morphological properties of neurons in LI of cortex

**Region**	**Age (day)**	**properties**	**Species &strain**	**Cell types**	**Reference**
SS	24–36	M & P	Rat (Wistar)	NGFCs, C-AC, FS, BS	[11]
Neocortex	0–10	Synaptic transmission	Mice	CR&Non-CR	[22]
Visual cortex	7–19	M&P	Rat (Wistar)	CR & NG	[23]
Frontal cortex	14–21	P	Rat (SD)	Not mentioned	[24]
Vision &SS	14–24	M&P	Rat (SD)	LS & nLS	[25]
Not mention	5–11	M&P	Rat (wistar)	CR	[26]
Vision cortex	adult	M	Cat	Not mentioned	[27]

M: morphological, P: physiological properties, SD: Sprague–Dawley, NG: Neurogliaform, SS: Somatosensory cortex, CR: Cajal-Retzius cells, NG: neurogliaform.

The anterior cingulate cortex (ACC) is a heterogeneous brain area which is involved in sexual attraction [28], fear memory [29-35] and chronic pain [3-5,36-42]. The change of cingulate glutamatergic synaptic transmission in chronic pain conditions has been intensively studied [3-5,36-42], It has been demonstrated that strengthening of synaptic transmission in the ACC were mediated by both pre- and post synaptic mechanisms [4,41]. And also previous work showed that the intrinsic properties of pyramidal neurons in layers II/III of the ACC were affected by peripheral nerve injury [43]. The inhibitory synaptic transmission is an import part of the neuronal network [2]. However, how the inhibitory components of cortical network change was less studied. Knowing the basic properties of LI neurons will help to understand the neuronal mechanism that mediated the functions of the ACC.

In the present study we investigated the intrinsic properties of LI neurons in the ACC of adult mice. Based on petilla classification scheme and unsupervised clustering results [1,44], LI neurons were classified as five groups, including: Spontaneous firing neurons; Delay-sAHP neurons, Delay-fAHP neurons, other neurons showed ADP were separated into two clusters, called ADP1 and ADP2. Pharmacological experiments showed that 2-amino-3-(5-methyl-3-oxo-1,2- oxazol-4-yl) propanoic acid (AMPA), kainate and N-Methyl-D-aspartate (NMDA) receptors were the major postsynaptic targets of glutamate, GABA_A_ receptors were the major targets for inhibitory synaptic transmission. To our knowledge, this is the first study to characterize LI neurons of the ACC from adult mice.

## Materials and methods

### Animals

Adult (8–10 weeks old) male C57BL/6 mice were purchased from Charles River. Mice were maintained on a 12 hr light/dark cycle. Food and water were provided *ad. libitum*. The Animal Care and Use Committee of University of Toronto approved all mouse protocols.

### Slice preparation

Male C57BL/6 mice were anaesthetized with isoflurane, the brain was then quickly removed and submerged in cold, oxygenated artificial cerebrospinal fluid (ACSF) containing the following (mM): 124 NaCl, 2.5 KCl, 0.5 CaCl_2_, 2 MgSO_4_, 25 NaHCO_3_, 1 NaH_2_PO_4_, and 10 glucose. Coronal slices (300 μm thick) containing the ACC were prepared using standard methods [37]. Slices were cut with a Vibratome section system (VT 1000), and transferred to submerged recovery chamber with oxygenated (95% O_2_ and 5% CO_2_) ACSF at room temperature for at least 1 hr.

### Whole-cell recordings

Experiments were performed in a recording chamber on the stage of a BX61WI microscope (Olympus) equipped with infrared differential interference contrast optics for visualization. The recording pipettes (2–3 MΩ) were filled with a solution containing (in mM) 120 K-gluconate, 5 NaCl, 1 MgCl_2_, 0.2 EGTA, 10 HEPES, 2 Mg-ATP, 0.1 Na_3_-GTP, 10 phosphocreatine disodium and 0.5% biocytin (sigma) (adjusted to pH 7.2 with KOH). Whole-cell recordings were performed at room temperature (24 ± 1°C) using a patch-clamp amplifier (multi-clamp 700B, Molecular Devices). The evoked post-synaptic response were recorded at 0.05 Hz, stimulations were delivered through a bipolar electrode which was put in layer III of the ACC. For synaptic response recordings, we used an internal solution containing (in mM): 120 Cs-gluconate, 5 NaCl, 1 MgCl_2_ 0.5 EGTA, 2 Mg-ATP, 0.1 Na_3_GTP, 10 HEPES, (adjusted to pH 7.2 with CsOH); 280–300 mOsm. Access resistance was 15–35 MΩ and was monitored throughout the experiment. Data were discarded if the access changed >10% during an experiment. Data were filtered at 2 kHz and digitized at 20 kHz using the digidata 1322A.

### Electrophysiological analysis

Different petilla terminologies [1] have been suggested to describe electrophysiological behaviors observed in cortical neurons, in our study, the following terminologies [20] will be used: Resting membrane potential (RMP) (1) was measured just after configuration of whole-cell patch; Input resistance (Rin) (2); and membrane capacitance (Cm) (3) were measured in voltage clamp mode. In current clamp mode, a 400 ms hyperpolarization current was used to evoke a voltage shift of 10–18 mV negative to resting membrane potential, and membrane time constant (Tau) (4) was determined after the fitting of this response to a single exponential function. Rheobase (Rho) (5) was the minimal current intensity which can generate at least one action potential (400 ms duration, 10 pA increments). The first spike latency (1^st^) (6) of the action potential (AP) was measured at Rheobase, as the time that the membrane voltage reached to the AP firing threshold (V_thre_) (7) which was defined as the first point on the rising phase of the spike at which the change exceeded 50 mV/ms. The amplitude of AP (8) was measured as the voltage difference between V_thre_ and the positive peak of AP. The spike width (9) was measured at half height of the total spike (measured from the V_thre_ level). The rise time (10) is the time that AP amplitude changes from 10% to 90%, while the decay time (11) is the time that AP amplitude decreases from 90% to 10%. In some cortical neurons, a complex waveform of afterhyperpolarization (AHP) has been recorded. The amplitudes (12, 13) and the latencies (14, 15) of the first (fAHP) and second (sAHP) components of AHP were measured as the difference between V_thre_ and the negative peak of the AHP. If an afterdepolarization (ADP) was present, its amplitude (16) and latency (17) was estimated as the difference between the negative peak of fAHP and the positive peak of ADP. For the firing pattern, the maximal firing frequency was defined as the last trace before the prominent reduction of action potential amplitude, the instantaneous frequency were fitted to:

(1) $${\text{F}}_{\text{sat}}={\text{A}}_{\text{sat}}\times {\text{E}}^{-\text{t}/\tau \text{sat}}+\text{t}*{\text{m}}_{\text{sat}}+{\text{F}}_{\text{max}}$$

where A _sat_ correspond to the amplitude of early frequency adaptation (18), τ_sat_ to the time constant of early adaptation (19), m_sat_ to the slope of the late adaptation (20) and F_max_ to the maximal steady state frequency (21). The amplitude reduction (22) and the duration increase (23) were further analysis on action potential discharges elicited by short pulses of depolarizing current (80 ms) in the 50–150 pA range. The amplitudes of the first two action potentials (A1 and A2) were measured from the threshold to the peak of the spike. Their durations (D1 and D2) were measured at half amplitude. The amplitude reduction and the duration increase were calculated according to (A1−A2)/A1 and (D2−D1)/D1, respectively [45].

### Visualization and imaging of the intracellular biocytin-labeled neurons

Slices containing biocytin-filled neurons were fixed overnight in a solution containing 4% paraformaldehyde at 4°C. Slices were then transferred into 0.01 M PBS for 1 hr and rinsed twice (5 min. each time) with PBS. Slices were then incubated in PBS with 0.1% Triton X-100 for a period of 2 hrs, followed by incubation with Cy3-conjugated streptavidin at 4°C for 6 hrs.

### Unsupervised clustering

Unsupervised clustering [46] was performed using the 23 physiological parameters, which was normalized to stander score, the Ward’s method was then performed to get the classification. The quality of the clustering was further quantified using silhouette analysis.

### Data analysis

Off-line analysis was performed using Clampfit 9. Sigmaplot 11.0 was used to plot and fit the data. Statistical comparisons were made using paired *t*-tests, one or two-way ANOVAs (Student-Newmann-Keuls test) were used for post-hoc comparison. SPSS 18.0 was used to do the cluster analysis. All data are presented as the mean ± S.E.M. In all cases, *P* < 0.05 was considered statistically significant.

## Results

### Whole-cell patch clamp recordings of neurons in LI of the ACC

**Figure 2:**
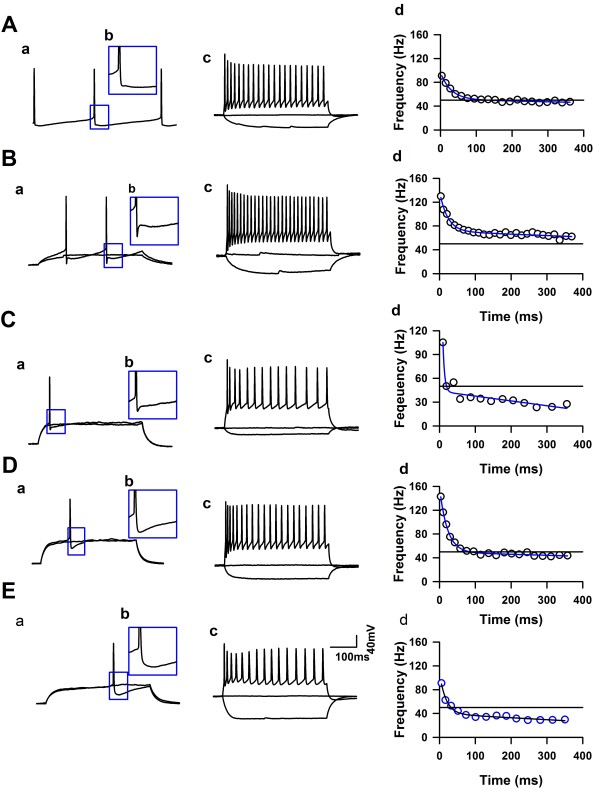
**The firing characteristics of spontaneous firing neurons. A** &**B**), The representative traces showed the spontaneous firing of APs from two neurons, respectively. **C** &**D**), The distributions of firing frequency collected from the represented neuron in Panel A and B, respectively. **E**), Distributions of the mean value and S.D. of firing frequency. **F**), The mean value of firing frequency negatively correlated with membrane time constant.

**Figure 3:**
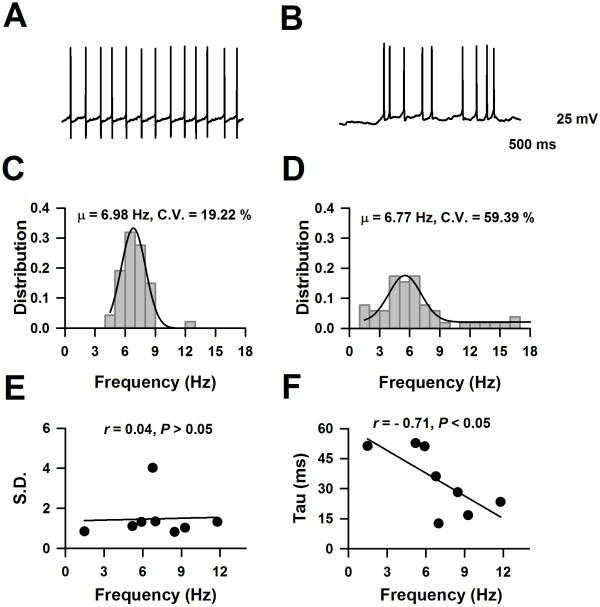
**Firing patterns of neurons in LI of the ACC from adult mice. A**), The representative showed one example of a spontaneous firing neuron in the LI of the ACC. **B**), The representative showed one example of d-AC neurons with fAHP in the LI of the ACC. **C**), The representative showed one example of d-AC neurons with sAHP in the LI of the ACC. Ca, Under subthreshold current stimulation, the membrane potential of this neuron developed a ramp depolarization. **D** &**E**), The representatives showed examples of neurons with ADP, named ADP1 and ADP2, respectively. a, the representative traces showed the change of membrane potential under the currents stimulation, the intensity of the currents were around the Rheobase of this neuron. b, The inset showed the AHP characteristics of the recorded neuron. c, Example traces showed the membrane potential change under currents stimulation. The APs fired at maximal firing frequency (for the definition, see the method part). d, the firing frequency changed under current stimulation, data was fitted by function: ${\text{F}}_{\text{sat}}={\text{A}}_{\text{sat}}\times {\text{E}}^{-\text{t}/\tau \text{sat}}+\text{t}*{\text{m}}_{\text{sat}}+{\text{F}}_{\text{max}}$. (For detail, see method part).

Whole-cell patch-clamp recordings were performed from neurons in LI and LII/III of the ACC from adult mice (Figure1B). After a stable recording was obtained, constant currents with different intensities were injected into the soma of neurons in the ACC. The intrinsic properties of the LI neurons were analyzed by using twenty-third electrophysiological parameters (see the method part). A total 71 LI neurons and 12 LII/III neurons (6 regular spiking, 2 intrinsic bursting and 4 intermediate neurons) were analyzed (n = 21 mice). In general, all the recorded LI neurons showed continuous firing and adaptation properties, expressed by the significant amplitude reduction and duration increase under short pulse of current stimulation, and also, both the amplitude and frequency adaptation of recorded LI neurons were obvious under maximal current stimulation. About 11% of recorded LI neurons (8/71) fired APs spontaneously (Figures 2 and 3A), which were classified as spontaneous firing neurons (SF). About 28% (20/71) of recorded neurons showed delay firing properties characterized by the generation of a ramp of membrane potential followed by delayed spiking (Figure3B & C) under constant current injections [25,47,48], which were classified as delayed adapting neurons (d-AC), the other LI neurons were called classical continuous adapting neurons (c-AC) (Figure3D & E) based on the petilla classification scheme [1]. About 44% of recorded LI neurons showed simple AHP, including fAHP (11/71) and sAHP (20/71), the others (40/71) showed afterdepolarization (ADP) components. The evoked synaptic responses were further recorded on 15 neurons of LI (n = 6 mice), and postsynaptic receptors that contribute to synaptic responses were further investigated by pharmacological approaches. 

### Two types of spontaneous firing characteristics

Eight (11%) recorded LI neurons fired APs spontaneously. As shown in Figure2A, this neuron fired APs continuously at about 7 Hz. The coefficient of variance (C.V.) of firing frequencies (Figure2A & C) was 19%, suggesting that APs fired at nearly constant frequencies; six neurons showed this firing characteristics. Figure2D & D showed another example, the average spontaneous firing frequency of this neuron was 7 Hz (from 1 to 19 Hz), the firing frequency showed Gauss distribution. The C.V reached 59%, which suggests a bigger variation of spontaneous firing frequency. Figure2E shows mean value distribution and S.D. of spontaneous firing frequencies; no relationship was found between the mean and S.D. of spontaneous firing frequency. We further analyzed the relationship between the mean value of the firing frequency and the membrane time constant; as shown in Figure2E, a negative correlation was revealed (*r* = −0.71, *P* < 0.05, Figure2F).

The electrophysiological properties of spontaneous firing neurons were further studied. As shown in Table2, spontaneous firing neurons had the highest input resistance, smallest membrane capacitance and highest resting membrane potentials compared to other groups. These observations suggest that the passive membrane properties of spontaneous firing neurons are different from other groups. To investigate the properties of APs, the membrane potentials were held at −60 mV, the amplitude, width and the first spike latency of APs were then determined. As shown in Table2, the AP amplitudes of SF neurons were similar to FS and continuous adapting neurons, but bigger than delay adapting neurons. Furthermore, SF neurons also had the lowest threshold for AP bursting, which may allow the neurons to fire more easily.

**Table 2:** Electrophysiological characteristics of LI neurons from adult mice (Ward’s)

	**SF**	**sAHP**	**fAHP**	**ADP1**	**ADP2**
	**(N = 4)**	**(N = 18)**	**(N = 11)**	**(N = 29)**	**(N = 6)**
Ri (MΩ)	1575.0 ± 103.1a	443.5 ± 37.8	421.3 ± 51.0	476.0 ± 44.5	413.3 ± 42.8
SF >> sAHP, fAHP, ADP1, ADP2					
C_m_ (pF)	18.2 ± 4.4a	44.0 ± 3.2	44.7 ± 4.2	47.0 ± 4.2	58.2 ± 8.9
sAHP, fAHP, ADP1, ADP2 >> SF					
Tau (ms)	42.0 ± 5.9	21.6 ± 1.1	18.5 ± 2.0	27.1 ± 2.1	39.3 ± 8.5
SF, ADP2 > sAHP, fAHP, ADP1					
RMP (mV)	−56.2 ± 5.1	−64.8 ± 1.7	−69.6 ± 1.4	−63.1 ± 1.3	−60.7 ± 2.9
SF > fAHP					
Rheobase (pA)	0	63.9 ± 10.4	71.8 ± 9.4	28.7 ± 4.1	30.8 ± 11.0
sAHP, fAHP > SF, ADP1, ADP2					
Amplitude (mV)	73.1 ± 7.3	63.2 ± 3.2c	80.9 ± 4.8	95.7 ± 2.4a	60.7 ± 3.7
ADP1>> SF, sAHP, fAHP, ADP2; fAHP > sAHP, ADP2					
First spike latency (ms)	192.6 ± 15.3	313.7 ± 28.7	229.0 ± 38.3	184.6 ± 15.1	240.4 ± 47.3
sAHP >> ADP1					
Amplitude of early frequency adaptation (Hz)	44.9 ± 7.3	75.3 ± 5.3	66.0 ± 9.9	99.7 ± 8.3a	50.9 ± 7.5
ADP1>> SF, sAHP, fAHP, ADP2					
Time constant of early adaptation (ms)	21.8 ± 4.9	25.5 ± 2.9	30.1 ± 3.7	23.5 ± 1.7	55.6 ± 11.3a
ADP2 >> ADP1, SF, sAHP, fAHP					
Slope of late adaptation (Hz/s)	−12.3 ± 5.4	−23.8 ± 4.6	−33.5 ± 6.7	−38.4 ± 4.5b	−4.5 ± 10.4
ADP2 > ADP1					
Maximal steady-state frequency (Hz)	41.7 ± 5.5	43.8 ± 2.4	48.9 ± 5.1	62.2 ± 4.0b	31.6 ± 3.3
ADP1 > ADP2					
Duration increase	27.7 ± 8.6	25.2 ± 2.6	20.3 ± 1.3	10.2 ± 1.0a	19.9 ± 4.7
SF, sAHP, fAHP, ADP2 >> ADP1					
Threshold (mV)	−52.5 ± 1.5a	−37.9 ± 1.4	−36.9 ± 1.2	−42.0 ± 0.8	−41.3 ± 1.1
sAHP, fAHP, ADP1, ADP2 >> SF; sAHP, fAHP > ADP1					
Half width (ms)	1.55 ± 0.14	1.76 ± 0.07c	1.34 ± 0.07	1.08 ± 0.03a	1.65 ± 0.04
SF, sAHP, fAHP, ADP2 >> ADP1; sAHP, ADP2 > fAHP					
Rise time (ms)	0.65 ± 0.03	0.69 ± 0.02	0.66 ± 0.02	0.59 ± 0.01b	0.76 ± 0.02
sAHP, fAHP, ADP2 > ADP1					
Decay time (ms)	1.67 ± 0.24	1.63 ± 0.08c	1.10 ± 0.10	0.78 ± 0.03a	1.48 ± 0.08
SF, sAHP, ADP2 > fAHP >> ADP1					

a, P < 0.01 compared to other groups, b, P < 0.05, compared to ADP2, c, P < 0.05 compared to fAHP.

### Unsupervised clustering of neurons in LI of the ACC

**Figure 4:**
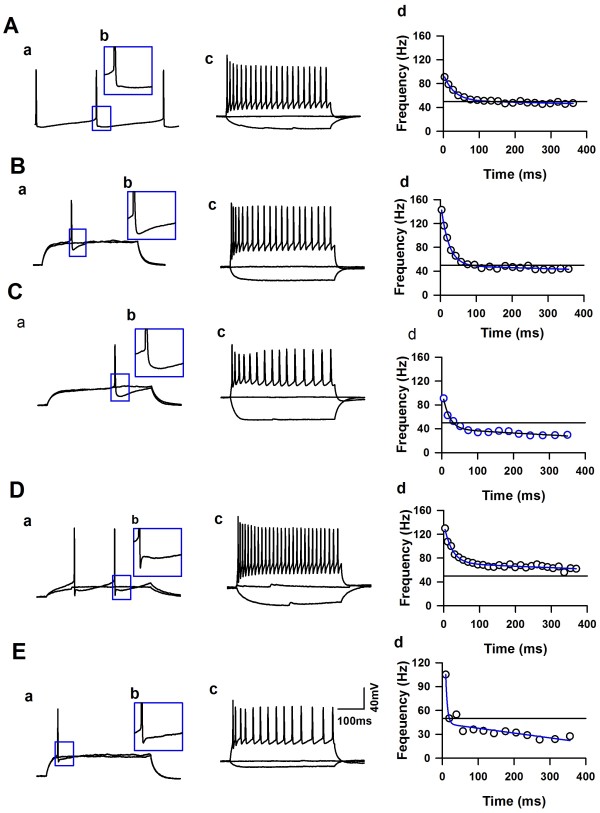
**Unsupervised clustering of neurons in LI of the ACC. A**), The classification of layer I neurons by using Ward’s method. **B**), Matching table of Ward cluster and cluster generated by k-means algorithm. **C** &**D**), The silhouette plot of the clusters by using Ward’s method and k-means clustering, respectively.

To classify the LI neurons, unsupervised clustering was performed by using the Ward’s method [20,46] on the recorded LI and LII/III neurons with 23 parameters. As shown in Figure4A, a total of 83 neurons were classified into six clusters. The 12 pyramidal neurons (6 regular spiking, 2 intrinsic bursting and 4 intermediate neurons) recorded within LII/III and 3 LI neurons (1 c-AC with sAHP, 2 c-AC with ADP) were put into one cluster (pyr) (Figure4A), the averaged silhouette width of this cluster was 0.14 ± 0.07 (−0.38 ~ 0.55, n = 15, Figure4C), suggesting the lower quality of this cluster. The majority of neurons with ADP were classified into two clusters, one cluster (ADP1) was consisted of c-AC neurons (26/43) and 3 SF neurons, the averaged silhouette width of this cluster was 0.45 ± 0.04 (−0.46 ~ 0.69), suggesting that the quality of this cluster is high. Another cluster (ADP2) was consisted of 4 c-AC neurons, 1 SF and 1 d-AC neuron, all the silhouette width from this group were positive (0.62 ± 0.07, 0.38 ~ 0.80, n = 6, Figure4C). The neurons with fAHP were classified into one cluster (fAHP), including 7 neurons from d-AC group and 4 neurons from c-AC groups, the silhouette width of this cluster distributed between 0.29 and 0.83. Four of SF neurons with sAHP were classified as one cluster (SF), the silhouette width of this cluster is high (0.79 ~ 0.91, Figure4C), suggesting that this cluster is very tight. The other neurons with sAHP were put into one cluster, including 12 d-AC and 6 c-AC neurons, this cluster was named as sAHP, three neurons within this cluster showed negative silhouette width (−0.15 ~ 0.69). 

Some of neurons showed negative silhouette width by using Ward’s method, this may be caused by the misassigning of neurons during the iterative process. To correct this point, we used the *k*-means algorithm to classify the recorded LI and LII/III neurons. As shown in Figure4B, about 85% neurons from Ward’s method were consistent with *k*-means results, 12 neurons were reassigned into different clusters in total (Figure4B). Seven regular spiking pyramidal neurons and one c-AC sAHP neurons originally assigned to the pyramidal neuron cluster were reassigned to the sAHP group, in the same way, three neurons originally from sAHP group were reassigned to ADP1 (two neurons) and ADP2 (one neurons), one neuron from Pyr and one from ADP1 were reassigned to ADP2. As shown in Figure4D, the reassignments change the silhouette width of neurons to positive, which suggest that the unsupervised clusters by *k*-means method were tight and with high quality.

### The electrophysiological characteristics of LI neurons

The unsupervised clustering results based on 23 electrophysiological parameters suggest that there were five groups of LI neurons. The SF group of neurons (n = 4) were characterized as highest input resistance, lowest membrane capacitance (Cm) and action potential firing threshold (Table2, Figure3A), suggesting that these spontaneously firing neurons have distinctive electrophysiological properties. About 41% of LI neurons formed the largest cluster (ADP1, n = 29, Figure3D) in our sample, these cells showed highest amplitude and narrowest half width of APs. The amplitude of early frequency of adaptation of this cluster was highest among groups, which suggest that these neurons had obviously frequency adaptation. However, the duration increase just reached to 10.2 ± 1.0% (n = 29), which was lower than other groups (Table2), suggesting the amplitude adaptation of these neurons was different. ADP2 cluster had highest time constant of early adaptation (Figure3E), except that, the major difference among fAHP (Figure3B), sAHP (Figure3C) and ADP2 were displayed by the shape of APs. When compared to neurons in the fAHP cluster, the neurons from sAHP cluster displayed lower amplitude of APs and broader half width (Table2).

### AMPA, kainate, and NMDA receptors are the major post-synaptic targets for excitatory synaptic transmission

**Figure 5:**
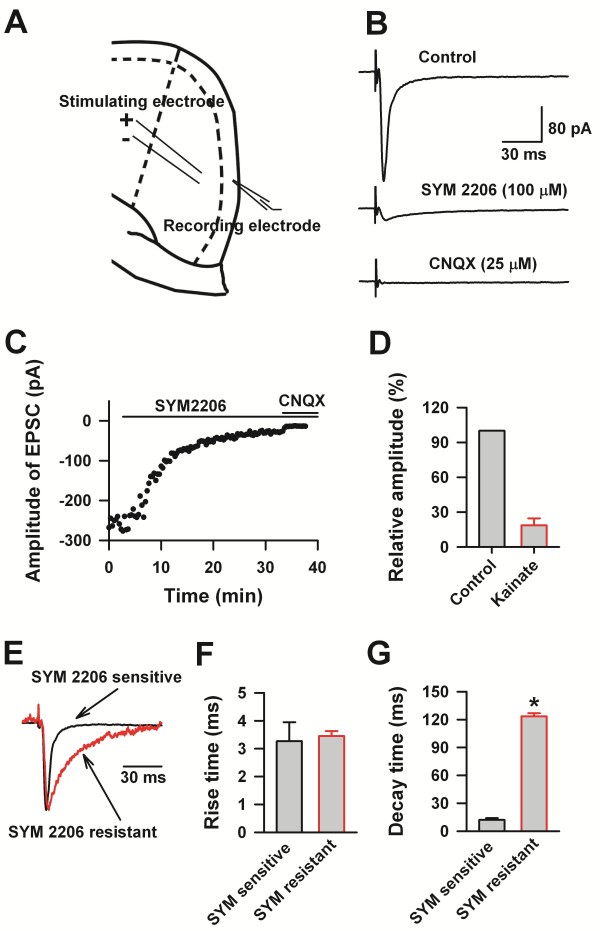
**AMPA and kainate receptor-mediated excitatory postsynaptic currents in LI neurons of the adult ACC. A**), Diagram showing the placement of stimulating and recording electrode in the ACC. **B**), The control represented evoked EPSCs recorded under the blocking of GABA_A_ and NMDA receptors activities with bath application of PTX (100 μM) and D-2-amino-5-phosphono-pentanoic acid (D-AP5, 50 μM). After application of SYM 2206 (100 μM), the residual currents were totally blocked by CNQX (25 μM). Each trace represented an average of 5–10 consecutive recording. **C**), Example showing the time course of SYM 2206 and CNQX effects on the neuron showed in **B**. **D**), The statistic data showing the percentage of SYM 2206 resistant-CNQX sensitive currents. **E**), Scaled traces showed the different kinetics of SYM 2206 sensitive and resistant currents. **F**), Pool data showing the rise time of SYM 2206 sensitive and resistant currents were similar. **G**), Summarized data showing the decay time of SYM 2206 sensitive and resistant currents were different. “*” indicate *P* < 0.05.

The connections between LI and other layers have been studied in mice and rats [10,11]. It was found that neurons in LI received inputs, and also projected inhibitory innervations to other layers, and therefore formed reciprocal connections [10]. However, the postsynaptic targets which mediated synaptic transmissions to LI neurons remain unclear. To investigate this point, we recorded the evoked synaptic responses at −60 mV on LI neurons by stimulating neurons in LII/III (Figure5A). After obtaining a stable baseline, we bath applied 100 μM SYM2206 (a selective AMPA receptor antagonist). Evoked responses decreased to 19 ± 6% of the baseline 30 minutes after SYM 2206 application (Figure5). We further applied CNQX (25 μM), an AMPA/Kainate receptors antagonist, the residual currents were totally eliminated 10 minutes after CNQX application. The rise time of the SYM2206 sensitive and resistant currents was 3.27 ± 0.68 and 3.45 ± 0.18 ms, respectively (Figure5). However, the decay times of the SYM 2206 sensitive currents were significantly shorter than the SYM2206 resistant components (*t* - test, n = 4, *P* < 0.05). Our data suggests that the currents recorded at −60 mV were mediated by AMPA and kainate receptors, and that these are therefore the major targets of glutamate mediated synaptic transmission in LI of the ACC. 

**Figure 6:**
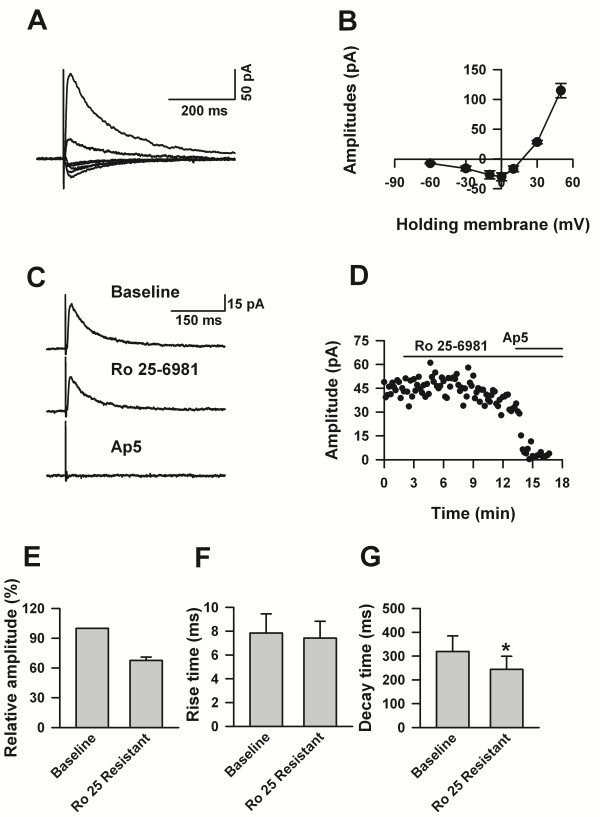
**NMDA receptor-mediated excitatory postsynaptic currents in LI neurons of the adult ACC. A**), The representative traces showing the NMDA receptors mediated currents at different membrane holding potentials. **B**), The summarized data showing the I-V curve of the NMDA receptors recorded from LI neurons of adult ACC. **C**), Sample traces showing the recorded NMDA receptors mediated currents in control, with Ro 25-6981, with D-AP5 solution. **D**), Example showing the time course of the effects of Ro 25-6981 and D-AP5 on the eEPSCs showed in C. **E**), Pool data showing the percentage of Ro 25-6981 resistant current. **F** &**G**), Statistic data showing that Ro 25-6981 did not change the rise time (F) but decreased the decay time (G) of the NMDA receptor mediated currents.

Previous studies have found that NMDA receptors are broadly expressed in the ACC, and are important for activity dependent synaptic plasticity [30]. To investigate whether NMDA receptors were involved in the excitatory synaptic inputs to LI, we pharmacologically blocked the activities of AMPA/kainate receptors and GABA_A_ receptors with the cocktail of CNQX and picrotoxin, and recorded evoked responses. As shown in Figure6, evoked EPSCs were recorded with a membrane holding at +30 mV, the recovery of currents was slow, represented by long decay time (Rise time:7.84 ±1.61 ms, decay time: 319.25 ± 65.37). The I-V curves showed an obvious Mg^2+^ blocking characteristic. Bath application of Ro 25-6981 (3 μM), a selective and potent blocker of GluN2B subunit containing NMDA receptors, decreased the amplitude of eEPSCs to 68 ± 3% (n = 5) of the baseline. Furthermore, Ro 25-6981 decreased the decay time of eEPSCs to 243.89 ± 55.13 ms (paired *t* - test, *P* < 0.05, n = 5), but had no effect on the rise time (Ro 25-6981: 7.41 ± 1.42 ms, paired *t* - test, *P* > 0.05, n = 5). The Ro 25-6981 resistant currents were delimited by AP5 (Figure6C&D). Therefore, our data suggests that NMDA receptors are involved in synaptic transmission from other layers to LI. 

### GABA_A_ receptors are the major inhibitory post-synaptic targets

**Figure 7:**
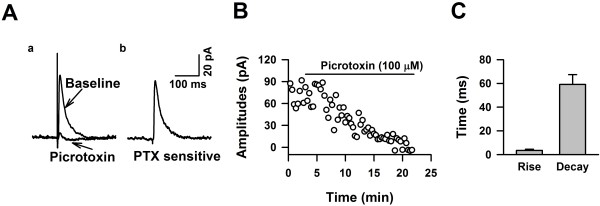
**GABA**_**A**_**receptor-mediated inhibitory postsynaptic currents in LI neurons of the adult ACC. A**), Sample traces showing that application of PTX blocked currents recorded at + 30 mV. **B**), Example showing the time courses of blocking effects of PTX on the evoked response represented in A. **C**), Summarized data showing the rise and decay time of the GABA_A_ receptor mediated currents recorded from LI neurons of the adult ACC.

To determine whether LI neurons receive GABAergic inputs from other layers, we recorded evoked responses by holding membrane potentials at +10 mV with CNQX perfusion, and under stimulation of LII/III, we recorded an outward current (Figure7A). After obtaining a stable baseline, we bath applied picrotoxin (PTX, 100 μM), a selective GABA_A_ receptor antagonist, and we observed that the recorded currents were reduced significantly and became inward currents 10 minutes after PTX application (Figure7D). The rise and decay time of the PTX sensitive currents was 3.58 ± 1.00 and 59.19 ± 10.34 ms, respectively (Figure7C). Our data therefore showed that LI neurons also receive GABAergic synaptic transmission from other layers.

## Discussion

Using whole-cell patch-clamp approach, we characterized neurons in LI of the ACC from adult mice. Based on the electrophysiological data and unsupervised clustering analysis, the neurons in LI can be classified into Spontaneous firing neuron; Delay-sAHP neurons, Delay-fAHP neurons, neurons with ADP were separated into two clusters, named ADP1 and ADP2, respectively. To our knowledge, this is the first report to characterize the intrinsic properties of neurons in LI of the ACC of adult mice. To further characterize the synaptic inputs to the LI neurons, we performed pharmacological experiments. The results showed that AMPA, kainate and NMDA receptors mediate excitatory synaptic transmission, while GABA_A_ receptors mediate the inhibitory synaptic inputs to LI neurons. Furthermore, this is the first report that adult LI neurons express kainate receptors.

### Different types of neurons in cortical LI

Here, we studied the firing properties of LI neurons from the ACC of adult mice (8–10 weeks). All the recorded neurons showed continuous firing properties, among them, 8 neurons fired APs spontaneously. Based on the firing patterns and petilla classification scheme [1], the others were classified as delay adapting and classical continuous adapting neurons, unlike Wozny C and SR Williams results from rats, we did not found the bursting neurons in LI of the ACC. Spontaneous firing neurons were characterized as displaying the highest input resistance, smallest membrane capacitance, and highest resting membrane potentials. To our knowledge, this is the first time to describe SF neurons in the cortical LI of adult mice. We further performed unsupervised clustering to classify the neurons in LI of the ACC by using different electrophysiological parameters. As shown in Figure3A, four of the SF neurons with sAHP were clearly classified as one group by Ward’s method, the silhouette values suggest that quality of the classification are high. The majority of the neurons with ADP were separated into two clusters by Ward’s method, the neurons of one cluster (ADP1) showed highest APs amplitude and narrowest half width and lowest duration increase, these properties were similar as that the FS interneurons recorded from LI (Wozny C and SR Williams), and LII-V of frontal cortex of rats [47,48]. However, the neurons in ADP1 in our sample also showed highest amplitude of early frequency adaptation, suggesting the frequency adaptation of these neurons was high, which was different from the classical FS neurons. Previous study showed that Late spiking neurons were major cell types in LI [25], here we found that about 28% of adult LI neurons exhibited late spiking characteristics in LI of the ACC, which were named as delay adapting neurons. Similar as previous report [47], the delay adapting neurons had widest spikes and most positive spike threshold compared to other types of neurons.

### Postsynaptic targets that mediate synaptic inputs to LI neurons

Previous studies have shown that GABA_A_, AMPA and NMDA receptors are the major targets of synaptic inputs to LI non-CR neurons in young rats [22]. Here we studied the receptors which mediated synaptic inputs to LI neurons through pharmacological interventions. In our experiment, about 81% of excitatory currents recorded at −60 mV were sensitive to SYM 2206, the rest were blocked by CNQX, which suggests that AMPA receptors are the major receptors which mediate excitatory synaptic input. Following the change of membrane potentials to +10 mV, we recorded another evoked PSCs which were blocked by PTX, indicating the involvement of GABA_A_ receptors to the synaptic inputs to LI. Our data showed that LI neurons received both excitatory and inhibitory inputs from other layers. By recording sEPSCs, we found that nearly all of the neurons in LI receive synaptic input (data not shown), which is inconsistent with previous work showing that neurogliaform cells, did not receive monosynaptic excitatory input from LII/III pyramidal neurons [11]. These inconsistencies may be a result of recording from different species, as well as the use of different recording methods. Since LI neurons also receive projections from thalamus [14,49], it is possible that some of the neurons in LI may just receive thalamic innervations, and therefore no responses can be recorded from LI neurons with the stimulation of neurons in LII/III.

About 19% of CNQX sensitive EPSCs were resistant to SYM 2206, which suggest that this current was mediated by kainate receptors. The percentage of kainate receptor mediated currents recorded on LI neurons was similar as that from the pyramidal neurons in LII/III of the ACC (SYM 2206 resistant, 19%) [50]. However, both the rise and decay time of the kainate receptors mediated currents were slower than that recorded from pyramidal neurons. This may come from the subunit component differences of the kainate receptors. It has been shown that the activities of GluK1 in LII/III of the ACC modulate GABA release [51], whether kainate receptors expressed on LI neurons display similar functions needs to be further studied. To our knowledge, this is the first report that cortical LI neurons of the adult moue express kainate receptors.

### NMDA receptors mediate the excitatory synaptic inputs to LI neurons

NMDA receptors play an important role in brain function and neuronal plasticity. Typically, functional NMDA receptor channels are heterotetrameric assemblies of two GluN1 subunits plus two GluN2 or GluN3 subunits; GluN2A-2D subunits provide the glutamate-binding sites and modifie channel properties such as current kinetics and channel conductance [52]. GluN2B subunit containing NMDA receptors have been detected in LII/III of the ACC [30]. Previously, Soda et al. reported that non-CR cells in LI expressed NMDA receptors [22]. Later on, Radnikow et al. found that GluN1/GluN2B subunit containing NMDA receptors were expressed by CR cells of LI on day 5-11 rats [26]. Here we showed that in LI of the adult ACC, neurons with GluN1/GluN2B subunit containing NMDA receptors mediated about 32% of the NMDA currents. Due to the lack of selective blockers for other subunits of GluN2, we were unable to investigate which subunit mediated the Ro 25-6981 resistant currents. The involvement of NMDA receptors to the plasticity of synaptic inputs in the CNS has been well demonstrated [53]. Previous studies have shown that GluN2B subunit of the NMDA receptor in the prefrontal cortex is critically involved in both contextual memory [30] and chronic pain [37,38]. Whether the GluN2B subunit of NMDA receptors is involved in the modulation of synaptic strengthen to LI neurons should be further studied.

### Functional role of LI neuron in ACC

Most of pyramidal neurons in the neocortex project apical tufts to LI, where they receive excitatory inputs from distant cortical areas or thalamus inputs [49,54]. Under synaptic stimulation, the apical dendrites generate Ca^2+^ spikes or NMDA spikes [55], which are important for the computation of pyramidal neurons [54,56] and synaptic plasticity [57,58]. LI neurons inhibit the apical dendritic Ca^2+^ spikes of layer V neurons, which are mediated by GABA_B1b_ receptors [59]; therefore LI neurons are involved in the modulation of synaptic plasticity received by pyramidal neurons. Different types of LI neurons may provide special inhibition to the tuft branches of pyramidal neurons [60]. For example, Spontaneous Firing L1 neurons may provide a long lasting inhibition to the targeted pyramidal neurons, and FS neurons may be involved into neuronal synchrony [61]. Whether periphery nerve injury or inflammation cause changes in the intrinsic properties of LI neurons needs to be further investigated.

The contributions of cortical over-excitability to chronic inflammatory and neuropathic pain have been well demonstrated [4]. LTP of synaptic transmission has been proposed as a cellular model for the chronic pain. In the ACC, it has been found that the synaptic transmissions were enhanced by periphery nerve injury or inflammation [36,40,62]. Through a combination of pharmacological approaches, we first identified that AMPA, Kainate and NMDA receptors mainly mediate the excitatory synaptic inputs to L1 neurons in the adult mice. Our previous studies have shown that the membrane expression of AMPA receptors was enhanced by nerve injury [36,41]. In addition, NMDA receptor NR2B subunit mediated responses were also enhanced in animal models of chronic inflammatory pain [37]. It is important to investigate possible changes of synaptic inputs to LI neurons of the ACC in chronic pain conditions in future studies. The present findings provide basic information for our understanding of cortical network and functions in physiological and/or pathological conditions such as learning & memory as well as chronic pain.

## Competing interests

The authors declare that they have no competing interests.

## Authors’ contributions

XYL, TC, and MZ conceived of the project and designed experiments. XYL and TC performed electrophysiological experiments. XYL, GD and KK analyzed data. XYL, GD and SQ wrote the manuscript. All authors read and approved the manuscript.
